# Regulating Histone Deacetylase Signaling Pathways of Myeloid-Derived Suppressor Cells Enhanced T Cell-Based Immunotherapy

**DOI:** 10.3389/fimmu.2022.781660

**Published:** 2022-01-24

**Authors:** Adeleye O. Adeshakin, Funmilayo O. Adeshakin, Dehong Yan, Xiaochun Wan

**Affiliations:** ^1^ Guangdong Immune Cell Therapy Engineering and Technology Research Center, Center for Protein and Cell-Based Drugs, Institute of Biomedicine and Biotechnology, Shenzhen Institutes of Advanced Technology, Chinese Academy of Sciences, Shenzhen, China; ^2^ University of Chinese Academy of Sciences, Beijing , China; ^3^ Department of Bone Marrow Transplantation and Cellular Therapy, St. Jude Children’s Research Hospital, Memphis, TN, United States

**Keywords:** MDSCs, HDAC, epigenetic signaling pathways, anti-PD-1/PD-L1, T cell-based immunotherapy

## Abstract

Immunotherapy has emerged as a promising approach to combat immunosuppressive tumor microenvironment (TME) for improved cancer treatment. FDA approval for the clinical use of programmed death receptor 1/programmed death-ligand 1 (PD-1/PD-L1) inhibitors revolutionized T cell-based immunotherapy. Although only a few cancer patients respond to this treatment due to several factors including the accumulation of immunosuppressive cells in the TME. Several immunosuppressive cells within the TME such as regulatory T cells, myeloid cells, and cancer-associated fibroblast inhibit the activation and function of T cells to promote tumor progression. The roles of epigenetic modifiers such as histone deacetylase (HDAC) in cancer have long been investigated but little is known about their impact on immune cells. Recent studies showed inhibiting HDAC expression on myeloid-derived suppressor cells (MDSCs) promoted their differentiation to less suppressive cells and reduced their immunosuppressive effect in the TME. HDAC inhibitors upregulated PD-1 or PD-L1 expression level on tumor or immune cells sensitizing tumor-bearing mice to anti-PD-1/PD-L1 antibodies. Herein we discuss how inhibiting HDAC expression on MDSCs could circumvent drawbacks to immune checkpoint inhibitors and improve cancer immunotherapy. Furthermore, we highlighted current challenges and future perspectives of HDAC inhibitors in regulating MDSCs function for effective cancer immunotherapy.

## Introduction

The tumor microenvironment is extremely immunosuppressive in the advanced cancer stage and targeting immunosuppressive phenotypes is a promising approach in cancer immunotherapy (1–4). The FDA approved two classes of immunotherapy for clinical use which include inhibitors of cytotoxic T-cell lymphocyte-associated protein 4 (CTLA-4) and programmed death receptor 1/programmed death-ligand 1 (PD-1/PD-L1) (5–7). Studies have shown that immunotherapy is effective in the treatment of certain cancers such as melanoma, lung, and renal carcinoma (6, 8–10). Nevertheless, only a few cancer patients respond to these treatments due to numerous factors such as tumor immunogenicity, inhibition of signal transduction, antigen presentation, upregulation of certain inhibitory molecules on T cells, poor persistence, and low effector function of T cells to demonstrate a cytotoxic effect on some tumor (11–15). Besides, tumor-infiltrating immunosuppressive cells such as myeloid-derived suppressor cells (MDSCs), tumor-associated macrophages (TAMs), regulatory T cells (Tregs), cancer-associated fibroblast (CAF) to mention a few contribute tremendously to the failure of immune checkpoint blockades (16–19). These immunosuppressive cells inhibit T cells effector functionality and their anti-tumor responses (16, 20).

In the tumor milieu, conventional-type 1 dendritic cells (DCs) possess the ability to cross-present tumor antigens and produce IL-12 to activate cytotoxic T cells for immune responses against cancer (21, 22). DCs are required to promote the anti-tumor effect of immune checkpoint blockades (22). More recently, NK and DCs subset (stimulatory DCs) axis were reported to define tumor response to checkpoint therapy, cytotoxic T cells response, and overall survival in melanoma tumor immune microenvironment (23, 24). Barry et al. demonstrated that a formative cytokine, Fms-related tyrosine kinase 3 ligand (FLT3LG) for conventional DCs was mainly produced by NK cells and played a critical role in regulating the level of stimulatory DCs for anti-tumor responses (23). Specifically, the authors showed that non-T cells have a significant impact on protective immunity since the frequencies of T cells exhaustion did not determine response to PD-1 therapy contrary to previous understanding (25). Thus, this observation requires further studies to delineate which immune cells predict responses to therapy.

Beyond the protective role of immune cells against tumor regression or elimination of pathogens, immune cells have been identified to play a critical role in normal tissue function such as tissue development and maintenance. Several immune cell types are heterogeneous which are distinct from the dual conception of tolerance versus destructive immunity. For instance, innate myeloid cells (DCs and macrophages) and T cells undergo multiple metabolic and epigenetic reprogramming impacting their roles in healthy or pathological conditions. This reprogramming can induce pro-inflammatory or anti-inflammatory cytokines production that drives contrasting activities of these immune cells. During chronic viral infection, epigenetic reprogramming leads to cytotoxic T cells exhaustion limiting T cells’ ability to recognize and kill non-self and infected cells (26). This exhaustion undermines the destructive potential of T cells responses and restricts the immunopathological effects for extensive eradication of infected host cells. Presently, other cell types that function with exhausted T cells to limit viral-specific T cell immunity are not fully characterized but are likely to be specific myeloid cells subsets (27). Tissue repair and wound healing is a good example of an immune response that is neither involved in tolerance nor destruction, but instead focuses on attaining tissue homeostasis. To achieve this, myeloid cell populations such as monocytes and macrophages have been identified (28). These emerging attributes of the immune system by engaging in non-destructive responses that promote cellular homeostasis besides pathogen protection were considered as a continuum between stringent approaches of tolerance and destruction regarded as immune accommodation archetypes (29). It is therefore evident that mobilizing the required immune response archetype is crucial for physiological and pathological conditions.

This necessitates the need to consider the immune system as a continuum of accommodation archetypes as these may influence our understanding of diseases especially cancer. As previously mentioned above, the tumor immune microenvironment accommodates several immune cell phenotypes that imitate these archetypes and contributes to tumor progression. Although, in some cancer types, data from patients’ cohorts exhibit wound healing gene signatures highlighting archetype remodeling (30). Another study showed variable components of late tissue-repair archetypes in cancers such as TAMs and Tregs (31). Krummel et al. highlighted that robust patient responsiveness to immunotherapies may require improved therapeutic or inhibition of subsets of certain immune archetypes in each tumor microenvironment (29). Thus, there is a need to explore how identifying archetype patterns will impact prognosis and immunotherapy for improved clinical responses in cancer patients.

MDSCs are pathologically activated immature myeloid cells that inhibit or induce several immune cells such as T, NK, Tregs, macrophages, neutrophils, and CAF during cancer, infection, graft versus host disease, and other conditions (32–35). MDSCs have been reported to demonstrate different roles in various pathological conditions (36). Most studies have studied MDSCs in the context of promoting immunosuppression in cancer, but recent studies have identified their therapeutic potential in reducing the severity of infection and autoimmune diseases which is yet to be fully understood (32, 37). Sarkar et al. showed that early recruitment of MDSCs subset in ocular herpes simplex virus type 1 (HSV1) infection suppressed effector CD4^+^ T cells proliferation and cytokine production in a contact-dependent manner (32). HSV1 infection initiates the manifestation of a severe inflammation called herpetic stromal keratitis (HSK) – a foremost cause of infectious blindness globally (38, 39). However, injection of *in vitro*-generated MDSCs from bone marrow precursor cells into HSV1-infected mice decreased the severity of HSK lesion at the onset of clinical HSK (32). Likewise, the transferred MDSCs in mice did not only induce anti-inflammatory responses but promoted endogenous Treg which could be clinically relevant (32).

In tumor-bearing mice, MDSCs can be characterized as CD11b^+^Gr1^+^ cells; these cells can be further subdivided into monocytic MDSCs (CD11b^+^Ly6G^-^Ly6C^hi^) and polymorphonuclear (PMN) MDSCs (CD11b^+^Ly6G^+^Ly6C^low^). MDSCs differentiate to other suppressive immune cells such as TAMs which accumulate in the TME and support tumor proliferation. Since MDSCs are phenotypically similar to monocytes and neutrophils, this led to the complexity in their identification and clearly defined functional assay. PMN-MDSCs account for about 70-80% of MDSCs in tumor models; secrete arginase 1 (ARG1) and upregulate NADPH which contributes to ROS production that inhibits immune cells function and activate of STAT3 signaling pathway (36, 40). On the other hand, M-MDSCs secrete ARG1, inducible nitric oxide (iNOS), and activate the STAT1 signaling pathway (36). Like murine MDSCs, there are two major subsets of human MDSCs which are M-MDSCs and PMN-MDSCs. In human peripheral blood mononuclear cell (PBMC), M-MDSCs consists of CD11b^+^CD14^+^HLA-DR^-/lo^CD15^-^ while PMN-MDSCs subset includes CD11b^+^CD14^-^CD15^+^ or CD11b^+^CD14^-^CD66b^+^. Recently, another subset of MDSCs in humans referred to as early-stage MDSCs (eMDSCs) was proposed to demonstrate colony-forming activity based on the immature nature of the cell population. eMDSCs is a mixed group of MDSCs with several immature progenitors that include – Lin^-^ (CD3, CD14, CD15, CD19, CD56) HLA-DR^-^CD33^+^ (41–43). However, these eMDSCs are yet to be identified or defined in mice.

MDSCs accumulate in patients’ tissues from several types of cancer (42–55). Reports have it that a higher frequency of tumor-infiltrating MDSCs is associated with advanced stage and high-grade tumors (52, 53). Importantly, several studies showed that the proportion of MDSCs in different cancer patients determines their responses to chemo- or immuno- therapy, and overall survival (51–54, 56–58). Presently, most immunotherapeutic strategies target lymphoid cells by adoptive transfer of tumor-specific T cells or reactivation of pre-existing anti-tumoral T-cells. Despite these approaches, certain drawbacks encountered with current therapies are associated with MDSCs accumulation. Therefore, researchers are investigating potential therapeutic strategies both at the pre-clinical and clinical levels aimed at targeting MDSCs for enhanced cancer immunotherapy.

Epigenetic modification in cancer cells had been identified over the years but its impact on immune cells regulation has only begun to emerge. A recent study proposed the combination of different epigenetic drugs as a promising anti-tumor therapy by blocking the expression of several members of the histone deacetylase (HDAC) family to alter the function of both PMN-MDSCs and M-MDSCs (59). In this way, targeting epigenetic pathways in cancer inhibited MDSCs’ role which may prime host immune responses for immunotherapy. More so, immune cell responses using epigenetic modifiers were reported in combination with other immunotherapies such as immune checkpoint inhibitors (60–62), adoptive cellular immunotherapy (63, 64), cytokine-based therapy (65), and vaccines (66). Therefore, future studies need to investigate the underlying mechanism(s) of how epigenetic agents can block MDSCs function for a potential anti-tumor effect that may guide translational research. Herein we summarize how manipulating HDAC expression in MDSCs could augment immune checkpoints blockade and highlight current challenges with HDAC inhibitors for effective cancer immunotherapy.

## Overview of Epigenetic Regulation of MDSCs

Epigenetic remodeling is a hallmark of cancer development and proliferation (67, 68). Epigenetic regulation is an inherent change to DNA that affects chromatin structure and gene expression without distorting the nucleotide sequence (69). Certain epigenetic therapies for cancer include HDAC, histone methyltransferase (HMT), and DNA methyltransferase (DNMT) inhibitors capable of stimulating tumor cells and enhancing host immune cells anti-tumor response. Treatment with epigenetic modifiers sensitizes response to immune checkpoint inhibitors in cancer patients (70). HMT inhibitors had been reported to be effective in the treatment of multiple myeloma (71) while DNMT inhibitors revealed promising outcomes in both pre-clinical and clinical studies available (72). Nevertheless, only a few HMT and DNMT inhibitors demonstrated anti-tumor potential in the clinic. On the contrary, HDAC inhibitors are a unique class of small molecule drugs with a wide range of effects on tumor cells and multiple cellular processes such as cellular differentiation, cellular compartmentalization, autophagy, and anti-angiogenesis (73, 74). Considering HDACs’ impact on chromatin structure, modulation of transcriptional factors, and their participation in multiple cellular processes, they are regarded as a promising molecular target to regulate gene expression and functions of specific proteins (75). The roles of HDAC inhibitors are not limited to tumor cells but have been identified to regulate immune cells’ function. Interestingly, recent studies reported that HDAC inhibitors reduced MDSCs function – a major immunosuppressive cell in the tumor microenvironment and promoted anti-tumor immune responses (59, 60, 76). However, it is yet to be fully deciphered the underlying mechanism of action on how HDAC inhibitors control MDSCs accumulation for improved cancer immunotherapy.

## HDACs Regulate MDSCs Function

Histone deacetylases (HDACs) are category of enzymes removing acetyl groups from N-acetyl lysine, an amino acid on histone tails to regulate chromatin structure and functions (77–79). They also modulate myriads of non-histone proteins (80). HDACs family has about 18 members which are classified into four (4) main classes: Class I, II, III, and IV (81). Class I, II, and IV are named classical HDACs and comprise 11 members while class III are homologs of yeast silent information regulator 2 proteins and referred to as sirtuins (81). Class I HDACs include HDAC - 1, 2, 3, and 8; class II HDACs include HDAC - 4, 5, 7, and 9 (class IIa) and HDAC - 6 and 10 (class IIb) whereas class IV only member is HDAC 11. Class I HDAC members are more abundantly distributed and well expressed in most cells without restriction to the nucleus alone (81). However, class II HDACs demonstrate certain restrictions with tissue-specific expression and alternates between the cytoplasm and nucleus (82).

Emerging evidence had shown that HDAC inhibitors possess an anti-tumor effect and demonstrated a synergistic effect with cancer immunotherapy (83). Nevertheless, the cytotoxic impact of HDAC inhibitors on tumor cells requires more understanding while little is known on how HDAC inhibition modulates immune cells function especially MDSCs. Several HDAC inhibitors affect MDSCs accumulation and function in contrasting ways as summarized in **Table 1**.

**Table 1:** Summary of the effects of HDAC inhibitors on MDSCs in several cancers.

HDAC Inhibitors	Class	Cancer type	Mechanism of action on MDSCs	References
Trichostatin A (TSA)	I, II	*In vitro*	Accumulation of CD11b+Gr1+ myeloid cell *via* iNOS1 and HO-1upregulation	(84)
Valproic acid	I	*In vitro*	Induced macrophage and DC generation	(85)
		Lymphoma	Reduced PMN-MDSCs accumulation *in-vitro* and decrease tumor growth *in-vivo* Repressed PD-L1, TLR4, Rb1, IL-4Rα/ARG1 signaling axis in MDSCs	(86)
			Decreased tumor-infiltrating MDSCs *via* repression of CCR2	(76)
		Melanoma	Induced M-MDSCs accumulation Downregulated MDSCs ARG1, IL-6 and IL-10 *via* IRF1/IRF8 activation	(60)
Entinostat	I	Lung Renal	Blocked MDSCs immunosuppressive function through reduced expression of ARG1, iNOS, and COX2	(87)
		Breast Pancreatic	Induced less suppressive PMN-MDSCs that promoted T cell proliferation	(88)
		Breast	Downregulation of CD40 expression in PMN-MDSCs and M-MDSCs	(89)
		Lung Breast Oesophageal	Reduced trafficking of PMN-MDSCs and MDSCs from bone marrow to pre-metastatic microenvironment *via* downregulating CXCR2 and CCR2.	(90)
		Lymphoma Lung	Reduced PMN-MDSCs immunosuppressive function	(59)
Ricolinostat	II		Reduced M-MDSCs accumulation	
Mocetinostat	I, IV	Colorectal	Reduced intratumoral MDSCs accumulation Induced expression of genes involved in immune evasion and antigen presentation	(91)
Sodium butyrate Vorinostat	I, II	*In-vitro*	Promoted MDSCs apoptosis *via* increased production of ROS *in vitro*	(92)
		Breast	Decreased MDSCs accumulation in blood, spleen, and tumor while activating CD8+T	
Vorinostat		Melanoma	Reduced MDSCs recruitment into the tumor site *via* downregulation of CCL2	(93)
		Neuroblastoma	Decreased M-MDSCs accumulation Reduced transcript for ARG1, S100A8, S100A9 and PD-L1	(94)
ACY241	IIb	Myeloma	Reduced MDSCs proportion	(95)
CG-745	I, IIb	Renal cell carcinoma Hepatocellular Carcinoma Colorectal	reduced Treg production *via* increased expression of IL-2 and IFN-γ Induced immune microenvironmental changes *via* Inhibiting tumor-infiltrating MDSCs	(96) (97)

## Trichostatin A

Trichostatin A (TSA), panHDAC inhibitor enhanced anti-tumor effect for Epstein-Barr virus (EBV)-associated tumor by inducing cell cycle arrest, apoptosis, and triggering EBV lytic cycle in lymphoblastoid cell lines (98). EBV-associated tumors are known to bypass immune surveillance while treatment with TSA-induced lytic genes that caused strong cytotoxic T lymphocyte responses (98, 99). Similarly, TSA suppressed proliferation and promoted apoptosis of esophageal squamous cell carcinoma *via* epigenetic regulation of apoptosis-related proteins (100). Besides, GM-CSF-induced bone-marrow-derived MDSCs in the presence or absence of TSA showed remarkable differences in myeloid cell differentiation *in vitro* (84). TSA promoted the accumulation of various undifferentiated myeloid cells exhibiting immunosuppressive functions like MDSCs in an iNOS1 and heme oxygenase-1 (HO-1) dependent manner. Likewise, an *ex vivo* experiment showed an increased proportion of CD11b^+^Gr1^+^ cells with suppressive activity in the spleen of naive mice treated with GM-CSF and TSA (84).

## Valproic Acid

On the contrary, a class I HDAC inhibitor, valproic acid (VPA) promoted the differentiation of *in vitro* GM-CSF induced bone marrow-derived-MDSCs into dendritic cells (DCs) and macrophages with less suppressive effect (85). Zhiqi et al., demonstrated that VPA reduced PMN-MDSCs accumulation from GM-CSF stimulated bone marrow cultured cells (86). They showed that VPA treatment in a dose-dependent manner attenuated the suppressive function of MDSCs on T-cells. It was reported that VPA attenuated the immunosuppressive function of MDSCs *via* downregulating the expression of retinoblastoma 1 (Rb1), toll-like receptor 4 (TLR4), programmed cell death 1 ligand (PD-L1), interleukin-4 receptor-alpha (IL-4Rα)/arginase axis signaling pathways. Similarly, VPA-conditioned *in vitro* derived MDSCs injected into EL4 tumor-bearing mice significantly inhibited tumor progression compared to the control mice (86). Furthermore, our group reported VPA treatment promoted the accumulation of less suppressive MDSCs mainly M-MDSCs in the spleen and bone marrow of B16F10-bearing mice with reduced IL-6, IL-10, and ARG1 expression *via* activation of IRF1/IRF8 transcriptional axis (60). Importantly, VPA treatment in bone marrow-derived MDSC co-culture with T cells reactivated T cells ability for TNFα production thus conferred anti-tumor effect (60). More recently, Zhiqi et al., revealed that VPA treatment of EL4-bearing mice reduced tumor-infiltrating M-MDSCs through downregulating CCR2 expression while there was no effect on PMN-MDSCs proportion (76). Although VPA did not affect both M-MDSCs and PMN-MDSCs accumulation in the spleen of EL4-bearing mice; T-cells proliferation was more when splenic PMN-MDSCs from mice administered VPA were co-cultured with T cells but no changes were observed on T cells proliferation in M-MDSCs isolated from VPA-treated mice compared to the control (76). Altogether these suggest the potential of VPA in reducing the immunosuppressive attribute of PMN-MDSCs with a slight effect on M-MDSCs to promote CD8^+^ T and NK cell proliferation and activation.

## Entinostat, Ricolinostat and 5-Azacytidine

Likewise, entinostat, another class 1 HDAC inhibitor promoted the accumulation of PMN-MDSCs and M-MDSCs in lung and renal murine tumor models (87). However, entinostat inhibited the immunosuppressive function of MDSCs *via* the reduced level of ARG1, iNOS, and COX2 as well as enhanced T cells proliferation in a co-culture system of MDSCs and T cells (87). In HER2/neu breast cancer and Panc02 metastatic pancreatic cancer murine model, entinostat reduced tumor burden and improved survival of the mice (88). It was reported that the anti-tumor effect of entinostat was through the accumulation of less immunosuppressive PMN-MDSCs in the TME that demonstrated impaired ability to inhibit T cells proliferation (88). Yusuke et al. reported that entinostat reduced PMN-MDSC and M-MDSCs proportion with downregulation of MDSC CD40 expression in metastatic estrogen receptor-positive breast cancer patients (89). Recently, Gabrilovich and colleagues demonstrated that treatment with entinostat in EL4 and LLC tumor models did not affect tumor growth (59). Although entinostat reduced PMN-MDSCs immunosuppressive function while M-MDSCs function was unaltered. It was observed that M-MDSCs had high expression of class II HDAC, specifically HDAC6 while further treatment with entinostat increased HDAC6 expression. Ricolinostat, a specific inhibitor of HDAC 6 reduced M-MDSCs accumulation without affecting tumor growth in mice while the combination of entinostat and ricolinostat significantly slowed tumor progression and reduce both MDSCs subsets in mice (59). These studies suggest that the anti-tumor effect of entinostat is cancer type-dependent and may need to be evaluated in other cancer types for an informed treatment option. Therefore, the combination of specific inhibitors of class I and II HDACs are required to block both MDSCs subsets accumulation and function for reduced tumor growth.

Recent reports demonstrated that MDSCs contributed to the development of pre-metastatic tumor microenvironment and residual tumor cells after surgical removal of the primary tumor (90, 101). While a low dose of entinostat (50nM) and 5-azacytidine (100nM) disrupted the pre-metastatic niche and inhibited metastasis. Mechanistically, it was deduced that this therapy restricted M-MDSCs and PMN-MDSCs trafficking from the bone marrow to the pre-metastatic microenvironment *via* downregulating CCR2 and CXCR2 expression respectively (90). Importantly, combined therapy of epigenetic modifiers and CCR2 antagonist increased disease-free survival as well as overall survival of mice. Entinostat and 5-azacytidine promoted the differentiation of splenic M-MDSCs into more –interstitial macrophage-like phenotypes, thus blocking MDSCs accumulation in the lung pre-metastatic niche (90).

## Mocetinostat

Mocetinostat is a selective inhibitor of class I and IV HDAC that regulates the epigenetic signaling of tumor and immune cells (102). In the CT26 colorectal mice model, it decreased intratumoral MDSCs and Treg accumulation while it increased CD8^+^T cells infiltration (91). Mocetinostat regulated histone modification and induced the expression of genes involved in immune evasion and antigen presentation in tumor cells (91). However, how mocetinostat controls MDSCs function remains unreported thus mechanistic studies on how mocetinostat impairs tumor-infiltrating MDSCs accumulation will be necessary.

## Vorinostat and Sodium Butyrate

Suberoylanilide hydroxamic acid, SAHA (also known as vorinostat), and Sodium butyrate (NaB) which are Class I and II non-specific HDAC inhibitors depleted accumulation of GM-CSF induced bone marrow-derived MDSCs and those isolated from the bone-marrow of 4T1 mammary-bearing mice (92). Treatment with SAHA and NaB promoted MDSCs apoptosis *via* increased production of ROS while *in vitro* generated bone marrow-derived MDSCs treated with SAHA and NaB failed to suppress T cells proliferation compared to control. Also, SAHA demonstrated its anti-tumor potential on the 4T1 mammary mice model by decreasing MDSCs accumulation in the spleen, blood, and tumor while promoting the activation and function of CD8^+^ T cells (92). Laura et al. showed SAHA reduced gene expression of pro-inflammatory cytokines (IL-1α, TNFα) and immunosuppressive growth factor (TGFβ) in tumor lysate from spontaneous ret transgenic mouse melanoma model. Also, chemokine (C-C motif) ligand 2 (CCL2) was downregulated which led to reduced MDSCs recruitment into the tumor site and contributed to reduced melanoma growth (93). In the neuroblastoma mice model, SAHA decreased M-MDSCs accumulation but increased the number of macrophage effector cells in TME (94). Importantly, the transcripts levels of arginase1, S100A8, S100A9, and PD-L1 which are critical for promoting immunosuppressive activities were significantly reduced in myeloid cells isolated from SAHA-treated tumors (94). Collectively, these studies suggest that SAHA creates an immune permissive tumor microenvironment and promises as a potential targeted therapy for various tumors.

## ACY241

HDAC6 specific inhibitor, ACY241 in combination with proteasome inhibitors and immunomodulatory drugs demonstrated anti-myeloma potential (95). It was reported that ACY241 reduces the proportion of MDSCs, Tregs, and the expression of PD-1/PD-L1 on CD8^+^ T cells in the bone marrow cells from myeloma patients. ACY241 induced antigen-specific memory T cells *via* the upregulation of transcription regulators such as Bcl-6, Eomes, HIF-1, and T-bet associated with the activation of downstream AKT/mTOR/p65 pathway (95). More recently, ACY241 induced accumulation of lung tumor-infiltrating T and NK cells while it reduced Tregs in non-small cell lung cancer (NSCLC)-bearing treated mice (103). Also, tumor-associated macrophages showed increased expression in MHC and co-stimulatory molecules such as CD80, CD86, and CD40 while it reduced inhibitory ligands like PD-L1 and PD-L2. ACY241 in combination with Oxaliplatin – a chemotherapy drug-induced T cells effector function, significant anti-tumor response, and increased survival of NSCLC bearing mice (103). This highlights the mechanisms by which ACY241 confers anti-tumor activity through regulating immune responses in patients and suggests a rationale for its clinical use in combination with other therapies in several cancers.

## CG-745

CG-745 is a class I and IIb HDAC inhibitor that has shown anti-cancer effects against prostate, colorectal, pancreatic, cholangiocarcinoma, and non-small cell lung cancer while its exact role in mediating immune responses remains unknown (104–107). In a murine model of renal cell carcinoma, CG745 reduced Treg production *via* increased expression of IL-2 and IFN-γ (96). A recent study demonstrated that CG-745 inhibited tumor-infiltrating M2 macrophage polarization and MDSCs while promoting NK and T cells proliferation in human PBMC (97). It was observed that CG-745 induced immune microenvironment changes and promoted PBMC cytotoxic activity.

HDAC 11, the newest and only class IV HDAC member was reported to be involved in the differentiation of bone marrow generated immature myeloid cells (iMC) to neutrophils, macrophages, and DCs (108). Bone marrow and spleen isolated from HDAC11 promoter-driven eGFP reporter transgenic mice (TgHDAC11-eGFP) showed high expression of eGFP denoting HDAC11 transcriptional activation in these cells at steady-state. When these mice were challenged with pancreatic cancer (PANCO2), MDSCs expansion was observed in their lymphoid tissues similar to tumor-bearing wild-type mice (108). Importantly, flow cytometry analysis revealed a reduction in eGFP expression of myeloid cells compartment from TgHDAC11-eGFP mice, indicating that the transition of iMC to MDSCs may require the downregulation of HDAC11. These authors further demonstrated that functional analysis using both TgHDAC11-eGFP and HDAC11KO mice strongly suggests that HDAC11 might be a negative regulator of MDSC expansion/function *in vivo* through control of suppressive IL-10 production. Despite the above observation myeloid-specific HDAC11 KO in tumor-bearing mice will be critical for understanding the role of HDAC11 in MDSCs accumulation and function.

## Effects of HDAC Inhibitors on Immune Checkpoint Proteins

Immune checkpoint proteins have continued to receive considerable attention to evaluate the potential of several treatment options for cancer immunotherapy. Anti-CTLA-4 therapy showed a better response in metastatic melanoma patients with a lower proportion of M-MDSCs in their peripheral blood compared to non-responders (109). This observation corroborates another study that reported higher M-MDSCs percentage on treatment with anti-CTLA-4 resulted in poor clinical response due to impaired T-cells activation and function (110). Other studies also reported the reduced proportion of circulating MDSCs level at onset as a prognostic marker for response to anti-CTLA-4 therapy in patients with malignant melanoma (53, 58, 111). CT26 colorectal carcinoma and 4T1 spontaneous mammary tumors shown to be modestly immunogenic and highly metastatic respectively are among the most common syngeneic tumors models used for evaluating novel therapeutic approaches. In CT26 and 4T1 resistant to ICB, treatment with epigenetic modulator decreased MDSCs accumulation and function, thereby improving tumor responses to anti-CTLA4 and anti-PD-1 therapy (112). Thus, combination therapy targeting MDSCs together with ICB improved tumor responses unlike monotherapy thus benefit cancer immunotherapy.

Surprisingly, it was observed that while entinostat significantly reduced MDSCs cell viability, 5-azacytidine had no effect (112). Another study showed that treatment of immune-resistance breast and pancreatic cancer cells with entinostat decreased PMN-MDSCs accumulation and their function that led to a less immunosuppressive tumor microenvironment (88). Interestingly, entinostat effect on MDSCs function and immune-related gene expression augmented response to anti-PD-1 and anti-CTLA4 therapy in both mice models (88). More recently, VPA plus anti-PD-1 antibody compared to their single therapy repressed the growth of B16F10 and EL4 tumor models *via* VPA impaired tumor-infiltrating M-MDSCs accumulation in the tumor microenvironment (76). These suggest that treatment with epigenetic modifiers inhibits MDSCs accumulation and function thereby augments immune checkpoint inhibitors for successful cancer treatment. Hence, the underlying mechanism of epigenetic regulators in immunobiology and how it affects the response to ICB needs to be fully investigated.

**Figure 1:**
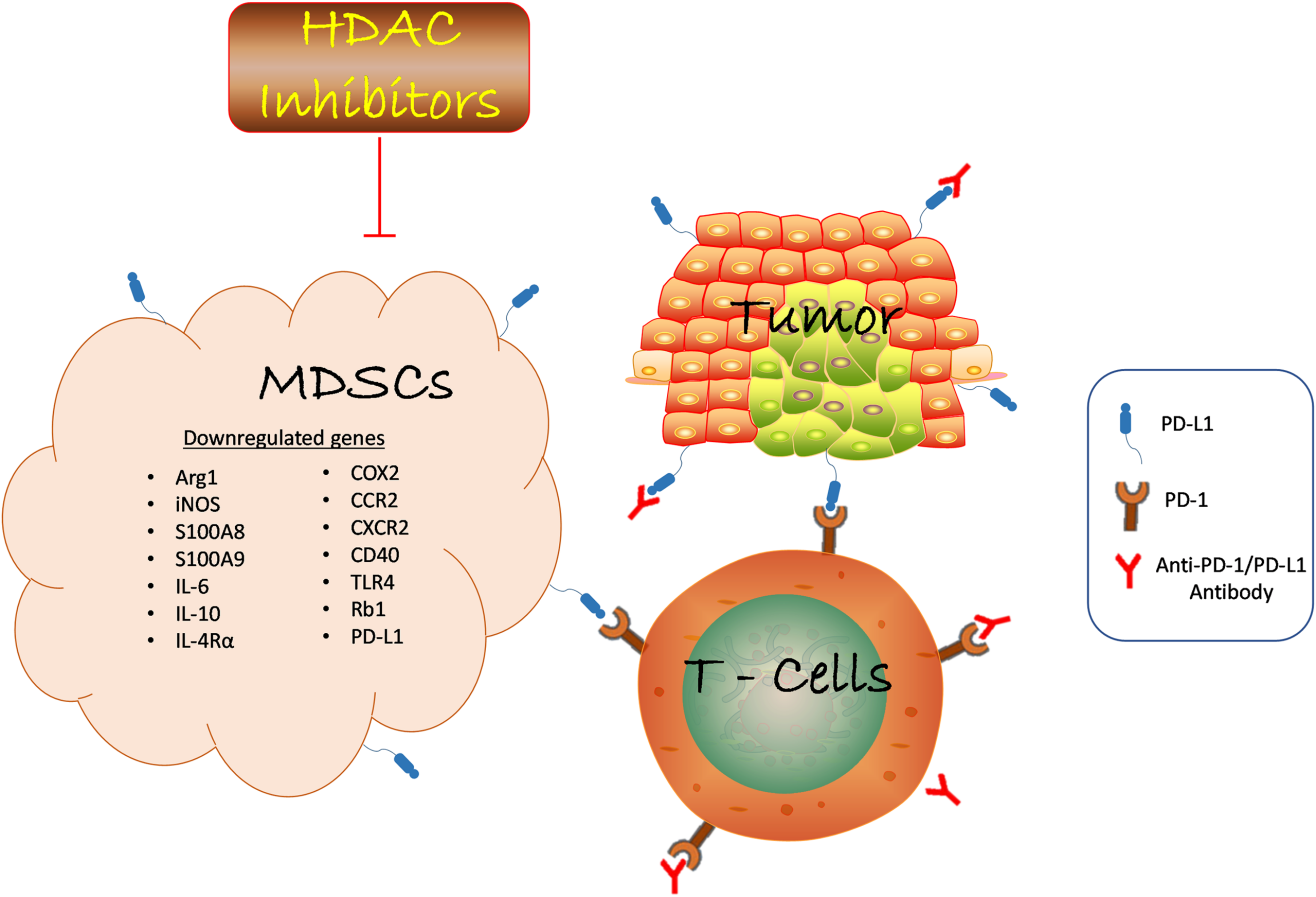
HDAC inhibition suppresses MDSCs function in the TME and promotes anti-PD-1/PD-L1 tumor immunotherapy. HDAC inhibition blocks tumor-infiltrating MDSCs accumulation in various cancer by downregulating the expression of genes involved in promoting the suppressive role of MDSCs which led to reduced tumor growth. Anti-PD-1/PD-L1 antibody inhibits immune checkpoint proteins expression on tumor and T-cell to confer anti-tumor effect. The combination of HDAC inhibitors and anti-PD-1/PD-L1 promotes T cells activation to inhibit tumor growth. Likewise, HDAC inhibitors augment anti-PD-1/PD-L1 tumor immunotherapy *via* reduced MDSCs function. Hence, the interaction of several immune cells within the TME determines the success of cancer immunotherapy strategies. HDAC, Histone deacetylase; MDSCs, Myeloid-derived suppressor cells; anti-PD-1/PD-L1, antibody against programmed death receptor 1/programmed death-ligand 1; ARG1, arginase 1; iNOS, inducible nitric oxide; IL-6, interleukin 6; IL-10, interleukin 10; IL-4Rα, interleukin 4 receptor alpha; COX2, cyclooxygenase 2; CCR, C-C Motif Chemokine Receptor 2; CXCR - CXC chemokine receptor 2; TLR4, toll-like receptor 4; Rb1, retinoblastoma 1.

In the tumor microenvironment, tumor and myeloid cells such as MDSCs, macrophages, and DCs can upregulate PD-L1 expression in response to inflammation (113, 114). This increased PD-L1 expression inhibits the effectiveness of cancer immunotherapy. Histone deacetylase (HDAC) inhibitors combat ICB resistance by attenuating the immunosuppressive function of MDSCs and sensitizing tumor cells to ICB. VPA and RGFP966 (HDAC 3 selective inhibitor) induced histone acetylation to facilitate PD-L1 transcription through the recruitment of bromodomain-containing protein 4 (BRD4) (115). Surprisingly, BRD4 inhibitor, JQ1 reduced PD-L1 upregulation triggered by HDAC inhibition. Inhibition of HDAC3 augmented the therapeutic effect of PD-L1 blockade by increasing PD-L1 expression on tumor and DCs in B-cells lymphoma (115). Furthermore, HDAC3 inhibition-induced PD-L1 expression could partly be one of the underlying mechanisms responsible for VPA resistance *via* evasion of immune surveillance checkpoints. This deduction is based on our previous study in which VPA alone failed to retard tumor growth in melanoma-bearing wild-type mice but slightly did in LLC-bearing mice (60). On the contrary, the combination of anti-PD-L1 antibody and VPA dramatically impaired tumor progression compared to PD-L1 blockade therapy alone. Mechanistically, MDSCs co-treated with VPA and anti-PD-L1 demonstrated impaired suppressive function and enhanced production of TNFα by T cells for anti-tumor effect. These findings corroborate the work of other researchers that host PD-L1 expression is crucial for PD-L1 blockade-mediated inhibition of tumor growth (114, 116). Thus, VPA could augment the therapeutic potential of the PD-L1 pathway blockade by increasing PD-L1 expression in tumor cells (**Figure 1**).

Recently, it was observed that bone marrow-infiltrating CD8^+^ T cells from acute myeloid leukemia (AML) patients demonstrated downregulated expression of immune checkpoint (IC) receptors including PD-1 which could contribute to upregulation of immune checkpoint ligands such as PD-L1 due to poor PD-1/PD-L1 interaction (117). However, treatment with VPA increased the expression of IC receptors. Likewise, genetic ablation of dual-specificity phosphatase 2 (DUSP2) (a newly identified T cell suppressor and key epigenetic immune modulator acting *via* HDAC complex) in CD8^+^ T cells upregulated genes involved in IC receptors. Interestingly, both VPA and DUSP 2 knockdown improved the effector functionality of CD8^+^ T cells; suggesting that downregulation in IC receptors is associated with pathological HDAC expression and resistance to IC inhibitors (117). Collectively, these studies depict HDAC inhibitors demonstrate the potential to increase immune checkpoint proteins expression and promote sensitivity to ICB as a combination therapy for ICB resistance in cancer patients.

## Future Perspectives and Current Challenges With HDAC Inhibitors in Cancer Immunotherapy

The majority of the FDA-approved HDAC inhibitors in the clinic are for the treatment of hematological cancers. Despite its clinical success for lymphoma and myeloma, it has failed to demonstrate significant effects as monotherapy in solid tumors. Although certain HDAC inhibitors such as entinostat (118), panobinostat (119), belinostat (120), and romidepsin (121) used as a single agent demonstrated significant anti-cancer effects in solid tumors from a phase I study but had negligible effects in phase II study. Besides, these inhibitors induced several side effects in the patients (122–126). Similarly, extensive pretreatment of the combination of HDAC inhibitors (azacytidine and entinostat) had an appreciable response in phase I/II study with recurrence and metastasis in non-small cell lung cancer (127).

To date, the reason HDAC inhibitors are efficacious in hematological malignancies unlike solid tumors is yet to be understood. However, several factors could be responsible such as lack of persistence and penetration into the solid masses as well as accumulation of immunosuppressive cells resident in solid tumors. Another critical and complex factor to consider in the administration of HDAC inhibitors is the metabolic state of the host; since epigenetic and metabolic changes in cancer cells are interrelated (128). Epigenetic modifiers such as HDACs regulate the expression of genes involved in metabolism and have become targets for cancer therapy (129). Although little is known on how regulating epigenetic or metabolic alteration could affect cancer immunotherapy and could be another future direction to explore.

Nevertheless, the future of HDAC inhibitors in solid tumors will depend tremendously on three major signs of progress in the field. One will be to improve the potency and specificity of next-generation HDAC inhibitors. Second, because HDAC inhibitors have reports of cellular toxicity profiles, it will be beneficial to understand the enigmatic HDAC biochemistry in cancer. This could reveal information on biomarkers that can be used to identify cancer patients that will respond to HDAC inhibitors therapy. Third, we believe that a comprehensive understanding of HDAC mechanisms of action will help identify other chemotherapies or ICIs that can be combined with HDAC inhibitors to circumvent current drawbacks. This will be a crucial landmark for HDAC therapies and will probably improve the clinical efficacy of future HDAC inhibitors.

Recently, it was discovered that female mounts a greater immune response compared to their male counterparts based on variation in sex hormones and sex-chromosome-related genes (130, 131). Conforti et al., reported that ICB was more effective in male patients compared to female patients while anti-PD-1/PD-L1 antibody combined with chemotherapy demonstrated enhanced therapeutic benefit for female patients compared to male patients (132). These suggested that therapies targeted at boosting immune responses will be less effective in female patients. On the other hand, phase III randomized clinical trials reported that sex-related factors may not affect the efficacy of ICB in melanoma patients (133). These contrasting results may be based on sample size or an inherent disparity in cancer etiology. Thus, gender-variation to immune response cannot be overemphasized in immunotherapy design and analysis. Since HDAC is well known to regulate mammalian gene expression, therefore, it is pertinent for other studies to investigate if HDAC inhibitors will augment anti-PD-L1 tumor immunotherapy or other ICB in both genders uniformly for effective translational research.

## Conclusion

Despite evidence from the literature that HDAC inhibitors are promising therapy to block MDSCs function in several cancers, it remains unknown the key molecular mechanisms by which HDACs specifically regulate MDSCs function – a major drawback to current cancer immunotherapies. While the data from *in vitro*-generated MDCSs are indispensable for *in vivo* studies, MDSCs obtained from tumor-bearing animals could differ in their suppressive properties and should be considered in future experimental designs. Therefore, it is pertinent for future studies to focus on elaborating how these emerging HDAC inhibitors in the clinic could completely block MDSCs accumulation or other immunosuppressive cells such as tumor-associated macrophages, regulatory T cells, or stromal cells resident in the tumor milieu.

## Author Contributions

AA and DY conceived the idea. AA and FA wrote the manuscript. DY and XW revised and supervised the writing. All authors contributed to the article and approved the submitted version.

## Funding

This work was supported by the National Key R&D Program of China (Grants 2019YFA0906100 and 2021YFC3300100), National Natural Science Foundation of China (Grants 82071772, 81501356, and 81373112), Key-Area Research and Development Program of Guangdong Province (2019B020201014), the Shenzhen Basic Science Research Project (Grants JCYJ201908 07161419228, JCYJ20170818155135838, JCYJ20170818164619194, and JCYJ20170413153158716), China Postdoctoral Science Foundation (2019M660220), Basic and Applied Basic Research Foundation of Guangdong Province (2019A1515110359), Nanshan pilot team project (LHTD20160004), Start-up funding (CYZZ20180307154657923), and the SIAT-GHMSCB Biomedical Laboratory for Major Diseases and Dongguan Introduction Program of Leading Innovative and Entrepreneurial Talents.

## Conflict of Interest

The authors declare that the research was conducted in the absence of any commercial or financial relationships that could be construed as a potential conflict of interest.

## Publisher’s Note

All claims expressed in this article are solely those of the authors and do not necessarily represent those of their affiliated organizations, or those of the publisher, the editors and the reviewers. Any product that may be evaluated in this article, or claim that may be made by its manufacturer, is not guaranteed or endorsed by the publisher.
